# Anti-IL-17 and Anti-IL-23 Therapies Modulate Serum Biomarkers of Intestinal Dysbiosis and Oxidative Stress Linked to Cardiovascular Risk in Patients with Psoriasis

**DOI:** 10.3390/life15111703

**Published:** 2025-11-03

**Authors:** Giuseppe Annunziata, Emanuele Scala, Laura Mercurio, Luca Sanna, Anna Dattolo, Gianluca Pagnanelli, Maria Grazia Lolli, Roberta Belli, Gaia Moretta, Silvia Savastano, Giovanna Muscogiuri, Maria Maisto, Roberto Ciampaglia, Vincenzo Piccolo, Gian Carlo Tenore, Cristina Albanesi, Stefania Madonna, Luigi Barrea

**Affiliations:** 1 Dipartimento di Psicologia e Scienze della Salute, Università Telematica Pegaso, Via Porzio, Centro Direzionale Isola F2, 80143 Naples, Italy; giuseppe.annunziata@unipegaso.it (G.A.); luigi.barrea@unipegaso.it (L.B.); 2 Laboratory of Experimental Immunology, Istituto Dermopatico dell’Immacolata, IDI-IRCCS, 00167 Rome, Italy; l.mercurio@idi.it (L.M.); l.sanna@idi.it (L.S.); a.dattolo@idi.it (A.D.); mg.lolli@idi.it (M.G.L.); r.belli@idi.it (R.B.); c.albanesi@idi.it (C.A.);; 3 Department of Dermatology, Istituto Dermopatico dell’Immacolata, IDI-IRCCS, 00167 Rome, Italy; g.pagnanelli@idi.it (G.P.); g.moretta@idi.it (G.M.); 4 Unità di Endocrinologia, Diabetologia ed Andrologia, Dipartimento di Medicina Clinica e Chirurgia, University of Naples Federico II, 80131 Naples, Italy; sisavast@unina.it (S.S.); giovanna.muscogiuri@unina.it (G.M.); 5 Centro Italiano per la Cura e il Benessere del Paziente con Obesità (C.I.B.O), University of Naples Federico II, 80131 Naples, Italy; 6 Cattedra Unesco “Educazione Alla Salute E Allo Sviluppo Sostenibile”, University of Naples Federico II, 80131 Naples, Italy; 7 Department of Pharmacy, University of Naples Federico II, 80131 Naples, Italy; maria.maisto@unina.it (M.M.); roberto.ciampaglia@unina.it (R.C.); vincenzo.piccolo3@unina.it (V.P.); giancarlo.tenore@unina.it (G.C.T.)

**Keywords:** psoriasis, TMAO, d-ROMs, oxLDL, inflammation, gut-skin axis, immune-cardio-metabolic modulation

## Abstract

Psoriasis is a chronic inflammatory skin disease whose pathogenesis involves not only cutaneous inflammation but also intestinal dysbiosis and oxidative stress (OxS). Monoclonal antibodies targeting interleukin (IL)-17 and IL-23 have demonstrated significant immunomodulatory effects; however, their impact on systemic parameters requires further investigation. We conducted a study on 33 patients with plaque psoriasis treated with anti-IL-17 or anti-IL-23 monoclonal antibodies. Dermatological parameters (Psoriasis Area and Severity Index (PASI) and Dermatology Life Quality Index (DLQI)), biomarkers of intestinal dysbiosis (trimethylamine-N-oxide (TMAO)) and OxS (reactive oxygen metabolites (d-ROMs) and oxidized LDL (oxLDL)) were evaluated. Anthropometric, metabolic, and adipose-derived hormonal parameters (adipokines) were also monitored. After 16 weeks of therapy, significant improvements were observed in PASI and DLQI scores (*p* < 0.001). TMAO levels were significantly reduced (*p* = 0.02), as were d-ROMs and oxLDL (*p* < 0.001). No significant changes were found in weight, body mass index, lipid profile, or adipokine levels (visfatin, leptin and adiponectin). Our data indicate that monoclonal antibody therapy not only improves psoriasis severity but also exerts beneficial effects on systemic biomarkers of dysbiosis and OxS, independent of metabolic or hormonal changes. These findings suggest a systemic mechanism of action, supporting a multifactorial therapeutic effect with potential implications for the prevention of cardiovascular risk.

## 1. Introduction

The introduction of monoclonal antibodies has transformed the treatment of systemic inflammatory diseases [1,2]. Engineered for enhanced bioavailability and target specificity, these agents are effective across autoimmune and chronic inflammatory disorders [1,2]. In dermatology, monoclonal antibodies targeting tumor necrosis factor alpha (TNF-α) and specific interleukins (ILs) are particularly effective in the treatment of psoriasis, given the central role of the IL-23/Th17 axis in the disease’s pathogenesis [3,4].

Psoriasis is a chronic immune-mediated skin disorder characterized by erythematous, scaly plaques caused by keratinocyte hyperproliferation and immune dysregulation [5,6]. Key cytokines, including TNF-α, IL-23, IL-17, and IL-22, drive persistent inflammation by orchestrating interactions among dendritic cells, Th17 lymphocytes, tissue-resident memory cells, and keratinocytes in a self-sustaining loop that promotes lesion development [4,5,6,7].

Beyond its cutaneous manifestations, psoriasis is increasingly recognized as a systemic condition associated with comorbidities such as psoriatic arthritis, obesity, and cardiovascular disease (CVD) [8,9,10]. Additionally, disruption of the intestinal barrier and microbiota dysbiosis have emerged as significant contributors, further amplifying systemic inflammation and metabolic dysregulation, which in turn increase the risk of CVD and other associated comorbidities in psoriasis patients [11,12,13,14]. Among microbiome metabolites, trimethylamine-N-oxide (TMAO)—produced by hepatic oxidation of trimethylamine from intestinal L-carnitine metabolism—emerges as a key biomarker of dysbiosis and a proatherogenic factor linked to inflammation and oxidative stress (OxS) [15,16,17,18]. Notably, elevated TMAO levels in psoriasis patients not only reflect disease severity but also serve as a potential marker for increased cardiovascular risk [18,19,20,21,22].

Despite increasing evidence of the role of gut dysbiosis and OxS in psoriasis, the effects of anti-IL-17 and anti-IL-23 therapies on these metabolites remain obscure. This study aims to evaluate the impact of anti-IL-17 and anti-IL-23 treatments on gut dysbiosis and systemic inflammation by measuring circulating levels of TMAO, oxidized LDL (oxLDL), and reactive oxygen metabolites (d-ROMs) before and after 16 weeks of therapy.

## 2. Materials and Methods

### 2.1. Study Design and Setting

This prospective pilot investigation involved patients with psoriasis and was carried out at the Dermatology Unit of IDI-IRCCS in Rome. The study period extended from July 2023 to July 2025. Approval was obtained from the Local Ethics Committee (Federico II Ethical Committee with protocol number n. 201/15 as well as Lazio Area 5 Committee of Rome with protocol number 721/CE/2024), and all procedures adhered strictly to the ethical principles outlined by the World Medical Association, particularly the Declaration of Helsinki governing research on human subjects. Study aims and procedures were explained in detail to all participants to ensure full understanding. Before enrolment, each participant provided written informed consent, confirming their voluntary agreement to participate.

### 2.2. Study Population

A total of 40 patients with moderate-to-severe plaque psoriasis were consecutively recruited from the Lazio region in Italy. Detailed medical histories were obtained for all participants. Inclusion criteria were: diagnosis of chronic plaque psoriasis, age ≥ 18 years; Psoriasis Area Severity Index (PASI) score ≥ 10, and body surface area (BSA) ≥ 10%. Patients with a baseline PASI < 10 but involvement of sensitive areas (scalp, hands, face, nails or genitals) were also included. Exclusion criteria included mild psoriasis treated topically, very severe psoriasis, use of systemic or biologic therapies for psoriasis within three weeks prior to enrollment, and severe psychiatric disorders.

**Figure 1:**
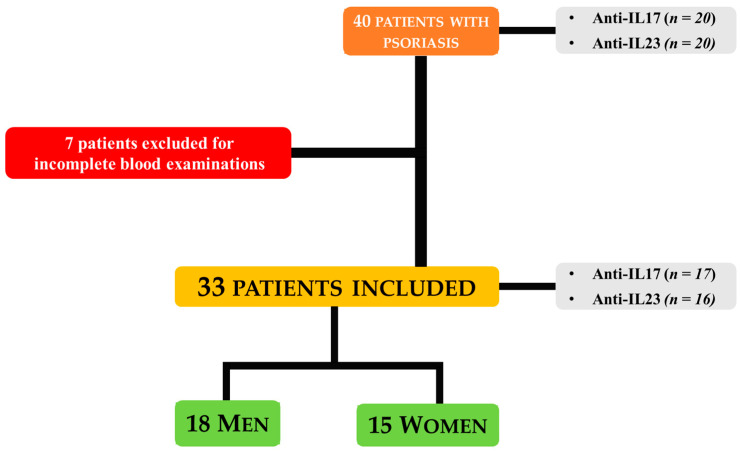
Overview of Patient Recruitment, Exclusion, and Final Cohort.

All enrolled patients initiated treatment with biologic drugs targeting either IL-17A (secukinumab, ixekizumab), IL-17A and IL-17F (bimekizumab), or IL-23 (guselkumab, risankizumab, or tildrakizumab) based on physician discretion and standard clinical practice. No randomization was performed. Biologics were administered according to Agenzia Italiana del Farmaco (AIFA) criteria using standard dosing regimens: secukinumab—300 mg; ixekizumab—160 mg at week 0, then 80 mg thereafter; bimekizumab—320 mg; guselkumab—100 mg; risankizumab—150 mg; tildrakizumab—100 mg (<90 kg) or 200 mg (>90 kg). All treatments were given as monotherapy without concurrent systemic or topical therapies. Of the 40 patients initially recruited, 7 were excluded from the analyses due to incomplete blood examinations, leaving a final sample size of 33 patients for all biomarker analyses. No separate control group was included; the study focused on evaluating systemic biomarkers in psoriasis patients before and after biologic therapy. The flowchart of patient selection and study inclusion is shown in Figure 1.

### 2.3. Psoriasis Assessment and Quality of Life Measures

Disease severity was assessed using both Psoriasis Area and Severity Index (PASI) and (Dermatology Life Quality Index) DLQI scores [23,24].

The PASI, a widely used gold standard for psoriasis severity, evaluates four anatomical regions—head and neck, upper limbs, trunk, and lower limbs—considering erythema, scaliness, and plaque thickness. Scores range from 0 to 72, with higher values indicating greater severity. PASI was recorded at baseline and at week 16 post-therapy. The primary endpoint was the pre-specified PASI75–100 response at week 16, defined as a 75%, 90%, or 100% improvement from baseline.

The DLQI was also measured at baseline and week 16. It comprises ten items grouped into six domains: symptoms and feelings (maximum 6 points), daily activities (maximum 6 points), leisure (maximum 6 points), work and school (maximum 3 points), personal relationships (maximum 6 points), and treatment (maximum 3 points). The total score ranges from 0 to 30, with higher scores indicating a greater impairment in quality of life (QoL).

### 2.4. Anthropometric Assessment

Anthropometric measurements were performed between 8:00 and 10:00 a.m. after an overnight fast, with participants in light clothing and barefoot. Body weight was measured to the nearest 0.1 kg using a calibrated beam scale, and height to the nearest 0.5 cm with a wall-mounted stadiometer. BMI was calculated as weight (kg) divided by height (m^2^) and classified according to WHO criteria for normal weight, overweight, and obesity (grades I–III) [25]. Waist circumference was measured to the nearest 0.1 cm at the natural waist crease, or if absent, at the midpoint between the lower rib margin and iliac crest, and evaluated against sex-specific cut-offs (>102 cm for males, >88 cm for females).

### 2.5. Blood Samples and Serum Analysis

Blood samples were collected from all participants in the morning after an overnight fast using serum separator tubes containing a clot activator and gel (BD Vacutainer). Samples were centrifuged at 1700× *g* for 20 min, after which serum was aliquoted and stored at −80 °C for subsequent analyses. On the same day, an aliquot of blood was used to determine lipid profile parameters, including total cholesterol, low-density lipoprotein (LDL-c), high-density lipoprotein (HDL-c), and triglycerides (TG). Parameters were classified according to standard clinical cut-offs: total cholesterol < 200 mg/dL, LDL-c < 100 mg/dL, HDL-c < 50 mg/dL for females and <40 mg/dL for males, and TG < 150 mg/dL.

Serum levels of the adipokines visfatin, leptin, and adiponectin were quantified using commercially available enzyme-linked immunosorbent assay (ELISA) kits (Visfatin: AG-45A-000-6Y TP-KIO1, Adipogen; Leptin: RD 191001100, BioVendor; Adiponectin: Human Total Adiponectin/Acrp30 Quantikine, R&D Systems), following the manufacturers’ instructions. Plates were read using an ELISA reader (Model 3550 UV; Bio-Rad, Hercules, CA, USA). The detection limits were 30 ng/mL for visfatin, 0.2 ng/mL for leptin, and 0.891 ng/mL for total adiponectin. Intra- and inter-assay coefficients of variation were <3% for all assays.

### 2.6. Determination of TMAO and OxS Biomarkers

Serum TMAO levels were measured by HPLC coupled with mass spectrometry (HPLC-HESI-MS/MS) following protein precipitation with methanol. Quantification was performed using a calibration curve, and the assay was validated for linearity, sensitivity, precision, and accuracy according to standard regulatory guidelines (Supplementary Tables S1 and S2). For the purpose of this study, TMAO levels were categorized into two groups based on the following threshold: TMAO levels < 5.94 µmol/L were classified as low, while TMAO levels > 5.95 µmol/L were considered high.

Serum ox-LDL and total oxidant capacity were assessed using the LP-CHOLOX assay and the d-ROMs Lab Test, respectively, with quality control measures to ensure reproducibility. For oxLDL, thresholds for classification were established as follows: normal (oxLDL < 1000 µEq/L), slightly high (1000–1500 µEq/L), moderately high (1500–2000 µEq/L), and very high (>2000 µEq/L). For d-ROMs, levels were classified as normal (≤300 UCARR), borderline (301–400 UCARR), and increasing OxS based on higher UCARR values.

All analyses were conducted in a single batch, in a randomized and blinded manner. Detailed protocols, including chromatographic and mass spectrometric parameters, calibration, validation procedures, and quality control data, are provided in the Supplementary Materials.

### 2.7. Statistical Analysis

Data distribution was assessed using the Kolmogorov–Smirnov test. Non-normally distributed variables were logarithmically transformed for analysis and back-transformed for presentation in tables. Continuous variables are reported as mean ± standard deviation (SD), frequencies as percentages, and pre- to post-treatment changes as Δ% with 95% confidence intervals (CIs). Differences between the two treatment groups at baseline was assessed using unpaired Student’s *t*-test, whereas paired-sample *t*-test was used to compare baseline and post-treatment values. A *p*-value < 0.05 was considered statistically significant.

Given the exploratory and pilot nature of this study, and the relatively small sample size (*n* = 33), no corrections for multiple comparisons were applied. The limited sample size may reduce the statistical power to detect small to moderate effects.

All analyses were performed using IBM SPSS Statistics software (PASW Version 21.0, SPSS Inc., Chicago, IL, USA).

## 3. Results

The initial study population consisted of 40 psoriatic patients (22 men and 18 women), 31 of whom had comorbidities such as type 2 diabetes (*n* = 7), hypercholesterolemia (*n* = 7), hypertriglyceridemia (*n* = 1), hypertension (*n* = 6), other cardiovascular diseases (*n* = 6), thyroid disorders (*n* = 3), and vitamin D deficiency (*n* = 1). These patients received 16 weeks of treatment with monoclonal antibodies targeting either IL-17 (anti-IL-17, *n* = 20) or IL-23 (anti-IL-23, *n* = 20). Treatment allocation was as follows: bimekizumab (*n* = 9), guselkumab (*n* = 3), ixekizumab (*n* = 8), risankizumab (*n* = 8), secukinumab (*n* = 3), and tildrakizumab (*n* = 9), based on physician discretion and clinical practice.

Of the initial 40 patients, 33 were included in the final analysis, as seven were excluded due to incomplete metabolic-lipid profile data. The final study cohort consisted of 33 patients (18 men and 15 women), divided into two treatment groups: anti-IL-17 (*n* = 17) and anti-IL-23 (*n* = 16). At baseline, the groups were comparable in terms of anthropometric and metabolic parameters, with lipid profiles largely within normal ranges (Table 1). However, the anti-IL-17 group exhibited a slightly higher prevalence of obesity and significantly elevated TMAO levels compared to the anti-IL-23 group (6.84 ± 6.51 vs. 2.88 ± 2.52, *p* = 0.03). Both groups demonstrated moderate-to-severe psoriasis, as evidenced by PASI values of 19.54 ± 6.91 in the anti-IL-17 group and 17.38 ± 8.05 in the anti-IL-23 group. Similarly, the impact on QoL, as assessed by the DLQI, was significant in both groups, with mean scores of 15.53 ± 8.09 and 15.50 ± 6.16, respectively, indicating a substantial impairment in daily functioning due to the disease.

To minimize individual variations and provide a clearer view of systemic changes, a pooled analysis was conducted to evaluate the overall response to 16 weeks of biological treatment targeting the IL-23/IL-17 axis. At week 16, no significant changes were observed in anthropometric parameters (body weight, waist circumference) or metabolic measures, including triglycerides, total cholesterol, LDL-c, HDL-c, and adipokines (visfatin, leptin, adiponectin), suggesting that the therapy had no substantial impact on these parameters during the study period. Percentage change (Δ%) and 95% CIs for each parameter are reported in Table 2.

From a dermatological perspective, treatment led to substantial improvements in both patient-reported outcomes and clinical measures. DLQI and PASI scores showed significant reductions from baseline (Δ%: −66.22 ± 27.38 for DLQI and −90.61 ± 22.17 for PASI; *p* < 0.001) (Table 3), with 95% CIs further supporting the robustness of these changes (7.80–12.20 for DLQI and 14.04–19.54 for PASI).

These changes translated into substantial gains in patients’ perceived QoL. The proportion of patients reporting a very large effect on QoL decreased from 39.4% to 12.1%, while those describing an extremely large effect dropped to zero, down from 30.3%. Conversely, the number of patients experiencing only a small effect increased from 3.0% to 36.4%, reflecting an overall enhancement in QoL. Reports of a moderate effect remained stable at 27.3%, and 24.2% of participants reported no effect after 16 weeks of treatment.

Reductions in disease severity paralleled these patient-reported benefits. Only 9.1% of patients remained in the PASI 10–15 range, down from 48.5%, and 6.1% were in the PASI > 15 range, compared with 30.3% at baseline. Most patients (18.2%) had PASI < 10, up from 21.2%, indicating a considerable improvement in clinical status (Table 3). These clinical gains were reflected in high rates of complete or near-complete resolution: PASI100 was achieved in 66.7% of patients (*n* = 22), PASI90 in 21.2% (*n* = 7), and PASI75 in 12.1% (*n* = 4) after 16 weeks of treatment.

Beyond clinical improvements, the analysis of biomarkers related to intestinal dysbiosis (TMAO) and OxS (d-ROMs and oxLDL) revealed significant reductions after 16 weeks of biologic treatment (Supplementary Figure S1). Specifically, TMAO levels decreased significantly (Δ%: −3.14 ± 81.17, *p* = 0.02), with a 95% CI of 0.58 to 3.11 (Table 4). Of note, the proportion of patients with high TMAO (>5.94 µmol/L) decreased from 21.2% to 12.1%, while those with lower TMAO levels (<5.94 µmol/L) increased from 78.8% to 87.9%. No significant changes in TMAO levels were observed within either the anti-IL-17 or anti-IL-23 groups before and after treatment.

Similarly, d-ROMs decreased significantly (Δ%: −18.19 ± 16.04, *p* < 0.001), with a 95% CI ranging from 54.35 to 120.98, reflecting reduced OxS. OxLDL levels also showed a significant decline (Δ%: −21.52 ± 20.51, *p* < 0.001), with a 95% CI of 15.47 to 707.29, indicating reduced lipid oxidation. The shift in OxS categories, from very high to normal levels, is detailed in Table 4.

## 4. Discussion

In this pilot study, we assessed the effects of anti-IL-17 and anti-IL-23 monoclonal antibodies on systemic biomarkers of dysbiosis (TMAO) and OxS (oxLDL, d-ROMs), alongside an analysis of lipid profile and adipokine levels in patients with moderate-to-severe psoriasis. Initially, 40 patients were enrolled and followed for 16 weeks after starting biologic therapy, with the final analysis conducted on 33 patients due to missing data.

In our cohort, many patients had comorbid metabolic conditions (e.g., type 2 diabetes, hypercholesterolemia, and hypertension) known to exacerbate psoriasis severity and complicate treatment outcomes [10,26,27]. Treatment allocation was based on physician discretion and clinical practice, reflecting real-world decision-making [26,28,29]. This variability in patient characteristics highlights the complexity of psoriasis management in clinical settings and underscores the importance of personalized treatment approaches.

The primary endpoint of the study was the pre-specified PASI 75–100 response at week 16, defined as a 75%, 90%, or 100% improvement from baseline. QoL was also assessed using the DLQI questionnaire. After 16 weeks of therapy, significant improvements were observed in both PASI and DLQI scores (*p* < 0.001), reinforcing the efficacy of IL-17 and IL-23 inhibitors [3,30,31] and supporting the critical role of the IL-23/Th17 axis in managing psoriasis lesions and improving QoL.

Furthermore, evidence suggests that biologic therapies targeting IL-17 and IL-23 may improve gut microbiota composition and potentially mitigate cardiovascular and metabolic risks by modulating inflammatory-related pathways [32,33,34,35,36]. However, IL-17 blockade continues to raise concerns due to its protective role in intestinal mucosal immunity [37,38]. In fact, anti-IL-17 therapies are associated with a relevant number of exacerbations and new onset of inflammatory bowel diseases (IBD) [39,40,41], while IL-23 inhibitors have proven effective in both Crohn’s disease and ulcerative colitis [42,43]. Despite these insights, the precise mechanisms by which biologics impact the microbiome in psoriasis remain unclear, warranting further studies to clarify these interactions. In 2024, Zhao et al. [33] observed that anti-IL-17 therapy (secukinumab) was associated with a trend toward normalization of the gut microbiome in 14 psoriasis patients, underscoring the complex interplay between this cytokine and the microbiota.

In our study, we observed a significant decrease in circulating TMAO levels following treatment with anti-IL-17 and anti-IL-23 therapies (*p* = 0.02). In particular, the anti-IL-17 group exhibited a higher prevalence of obesity and significantly elevated TMAO levels compared to the anti-IL-23 group (6.84 ± 6.51 vs. 2.88 ± 2.52, *p* = 0.03). This aligns with existing literature linking TMAO to obesity [25,44], suggesting its potential as a biomarker for both systemic inflammation and metabolic dysfunction. However, we did not observe any significant changes in BMI, lipid profiles, or adipokine levels (including visfatin) following biologic treatment. Notably, in a previous study of ours [45], anti-IL-23 therapy was found to modulate visfatin levels after 16 weeks in overweight women (N = 66). The lack of significant adipokine modulation in the current study may be due to the small sample size, and the complex regulation of adipokines in psoriasis [10]. Further stratified analyses (e.g., by sex, clinical characteristics, or specific therapy) may be necessary to gain a deeper understanding into these relationships.

The concomitant reduction in oxLDL and d-ROMs provides further evidence that IL-17 and IL-23 blockade may exert indirect cardioprotective effects. Elevated oxLDL levels in psoriasis contribute to endothelial dysfunction and atherosclerosis [46,47,48,49,50]. Mechanistically, oxLDL induces endothelial activation through the lectin-like oxLDL receptor, triggering pro-inflammatory signaling cascades [51,52]. Additionally, oxLDL has been shown to stimulate IL-23 secretion at the vascular wall, creating a pathogenic feedback loop between OxS and cytokine-mediated inflammation [53]. Therefore, by inhibiting IL-17 and IL-23, biologic therapies may disrupt this loop, reducing vascular OxS and preventing endothelial dysfunction.

Our study’s strengths include the use of well-established biomarkers and the prospective intra-subject design, which minimizes inter-individual variability. However, several limitations should be acknowledged. The small sample size and pilot nature of the study limit the generalizability of the findings. Additionally, the absence of a control group prevents conclusions about causality, while the short follow-up period precludes long-term evaluation of treatment effects. Furthermore, kidney function data were not collected, and confounding factors such as diet and concomitant medications were not controlled. Larger studies with longer follow-up and control for these factors are needed to confirm the findings. Importantly, without direct microbiome profiling, it is not possible to definitively attribute the observed changes in TMAO levels to microbial alterations.

## 5. Conclusions

This study provides new insights into the systemic effects of biologic therapies targeting IL-17 and IL-23 in psoriasis. Our findings suggest that these therapies not only improve skin lesions but also reduce circulating biomarkers associated with gut dysbiosis and OxS, potentially mitigating metabolic and cardiovascular risks. The ability of these biologics to influence microbiota and oxidative processes open new avenues for understanding the complex nature of psoriasis. Future research should focus on prospective multicenter validation of these findings and further exploration of microbiome correlations. Integrating immunological, microbiological, and metabolic data will be crucial for providing a more holistic understanding of psoriasis and informing therapeutic strategies to address both cutaneous and systemic aspects of the disease.

## Figures and Tables

**Table 1:** Baseline characteristics of psoriatic patients by treatment group (*n* = 33).

Parameters	Anti-IL-17 (*n* = 17)	Anti-IL-23 (*n* = 16)	*p*-Value
Males, *n* (%)	9 (52.90%)	9 (56.30%)	-
Age, years	46.41 ± 16.85	50.12 ± 19.96	0.57
Weight, kg	86.02 ± 18.72	77.37 ± 19.44	0.20
BMI, kg/m^2^	30.53 ± 6.75	27.94 ± 6.43	0.27
WG, cm	100.81 ± 17.13	92.59 ± 18.28	0.19
Triglycerides, mg/dL	124.29 ± 53.03	116.06 ± 60.36	0.68
Total cholesterol, mg/dL	181.65 ± 38.44	190.62 ± 33.57	0.48
HDL-c, mg/dL	59.47 ± 15.80	57.62 ± 13.76	0.72
LDL-c, mg/dL	100.06 ± 31.53	111.26 ± 30.96	0.31
TMAO, µmol/L	6.84 ± 6.51	2.88 ± 2.52	0.03
d-ROMs, UCARR	405.59 ± 122.88	437.87 ± 140.63	0.49
oxLDL, µEq/L	848.31 ± 252.30	1261.02 ± 1354.09	0.25
Visfatin, ng/mL	5.79 ± 3.85	5.59 ± 3.44	0.89
Leptin, pg/mL	61.29 ± 71.30	42.04 ± 37.72	0.41
Adiponectin, µg/mL	6.61 ± 3.65	9.57 ± 5.91	0.13
PASI	19.54 ± 6.91	17.38 ± 8.05	0.42
DLQI	15.53 ± 8.09	15.50 ± 6.16	0.99

Data are presented as mean ± SD or *n* (%). Baseline differences between groups were assessed using unpaired Student’s *t*-test. *p* < 0.05 was considered statistically significant. Abbreviations: BMI, body mass index; WG, waist girth; HDL-c, high-density lipoprotein cholesterol; LDL-c, low-density lipoprotein cholesterol; TMAO, trimethylamine N-oxide; d-ROMs, reactive oxygen metabolites; oxLDL, oxidized LDL; PASI, Psoriasis Area Severity Index; DLQI, Dermatology Life Quality Index.

**Table 2:** Pooled Analysis of Anthropometric and Metabolic Parameters in Psoriatic Patients (*n* = 33) Treated with Anti-IL-17 or Anti-IL-23 Therapy at Baseline and After 16 Weeks.

Parameters	Baseline	Week 16	*p*-Value	Δ%	95% CI
Males, *n* (%)	18 (54.5%)	-	-	-	-
Age, years	48.21 ± 18.23	-	-	-	-
Weight, kg	81.83 ± 19.28	81.88 ± 19.45	0.88	0.02 ± 2.42	−0.69–0.59
BMI, kg/m^2^	29.27 ± 6.62	29.30 ± 6.73	0.85	0.02 ± 2.42	−0.27–0.22
WG, cm	96.83 ± 17.91	96.91 ± 18.24	0.77	0.04 ± 1.82	−0.67–0.50
Triglycerides, mg/dL	120.30 ± 55.96	121.12 ± 57.12	0.95	3.92 ± 29.99	−14.13–12.50
Total cholesterol, mg/dL	186.00 ± 35.89	187.85 ± 42.15	0.69	1.30 ± 13.98	−11.21–7.51
HDL-c, mg/dL	58.58 ± 14.64	57.33 ± 16.45	0.36	−1.39 ± 15.80	−2.19–4.68
LDL-c, mg/dL	105.49 ± 31.28	102.41 ± 34.23	0.56	−0.79 ± 24.40	−7.45–13.60
Visfatin, ng/mL	5.68 ± 3.56	4.26 ± 3.80	0.11	−31.78 ± 46.46	−0.34–3.18
Leptin, pg/mL	50.93 ± 55.43	53.34 ± 55.15	0.55	59.01 ± 284.19	−11.60–6.78
Adiponectin, µg/mL	8.20 ± 5.13	8.29 ± 6.18	0.89	6.42 ± 46.91	−2.10–1.93

Data are mean ± standard deviation (SD) or *n* (%). Paired-sample Student’s *t*-test was used to compare baseline and post-treatment values. *p* < 0.05 was considered statistically significant. Δ% indicates the percent change from baseline to post-treatment, while 95% CI represents the confidence interval of the change. Abbreviations: BMI, body mass index; WG, waist girth; HDL-c, high-density lipoprotein cholesterol; LDL-c, low-density lipoprotein cholesterol.

**Table 3:** Pooled Analysis of Dermatological characteristics in Psoriatic Patients (*n* = 33) Treated with Anti-IL-17 or Anti-IL-23 Therapy at Baseline and After 16 Weeks.

Parameters	Baseline	Week 16	*p*-Value	Δ%	95% CI
DLQI	15.51 ± 7.11	5.51 ± 5.78	<0.001	−66.22 ± 27.38	7.80–12.20
No effect on QoL, *n* (%)	-	8 (24.2%)			
Small effect on QoL, *n* (%)	1 (3.0%)	12 (36.4%)			
Moderate effect on QoL, *n* (%)	9 (27.3%)	9 (27.3%)			
Very large effect on QoL, *n* (%)	13 (39.4%)	4 (12.1%)			
Extremely large effect on QoL, *n* (%)	10 (30.3%)	-			
PASI	18.49 ± 7.44	1.70 ± 3.74	<0.001	−90.61 ± 22.17	14.04–19.54
<10, *n* (%)	7 (21.2%)	6 (18.2%)			
10–15, *n* (%)	16 (48.5%)	3 (9.1%)			
>15, *n* (%)	10 (30.3%)	2 (6.1%)			

Data are mean ± standard deviation (SD) or *n* (%). Paired-sample Student’s *t*-test was used to compare baseline and post-treatment values. *p* < 0.05 was considered statistically significant. Δ% indicates the percent change from baseline to post-treatment, while 95% CI represents the confidence interval of the change. Abbreviations: DLQI, Dermatology Life Quality Index; PASI, Psoriasis Area and Severity Index; QoL, quality of life.

**Table 4:** Pooled Analysis of TMAO and OxS Parameters in Psoriatic Patients (*n* = 33) Treated with Anti-IL-17 or Anti-IL-23 Therapy at Baseline and After 16 Weeks.

Parameters	Baseline	Week 16	*p*-Value	Δ%	95% CI
TMAO, µmol/L	4.92 ± 5.31	3.07 ± 2.60	0.02	−3.14 ± 81.17	0.58–3.11
<5.94, *n* (%)	26 (78.8%)	29 (87.9%)			
>5.95, *n* (%)	7 (21.2%)	4 (12.1%)			
d-ROMs, UCARR	421.24 ± 130.72	333.58 ± 96.54	<0.001	−18.19 ± 16.04	54.35–120.98
Normal, *n* (%)	3 (9.1%)	11 (33.3%)			
Borderline, *n* (%)	4 (12.1%)	2 (6.1%)			
Low OxS, *n* (%)	2 (6.1%)	7 (21.2%)			
Middle OxS, *n* (%)	9 (27.3%)	7 (21.1%)			
High OxS, *n* (%)	4 (12.1%)	-			
Very high OxS, *n* (%)	11 (33.3%)	6 (18.2%)			
oxLDL, µEq/L	1048.41 ± 967.05	687.03 ± 187.26	<0.001	−21.52 ± 20.51	15.47–707.29
Normal, *n* (%)	3 (9.1%)	10 (30.3%)			
Slightly high, *n* (%)	14 (42.4%)	17 (51.5%)			
Moderately high, *n* (%)	7 (21.2%)	4 (12.1%)			
Very high, *n* (%)	9 (27.3%)	2 (6.1%)			

Data are mean ± standard deviation (SD) or *n* (%). Paired-sample Student’s *t*-test was used to compare baseline and post-treatment values. *p* < 0.05 was considered statistically significant. Δ% indicates the percent change from baseline to post-treatment, while 95% CI represents the confidence interval of the change. Abbreviations: TMAO, trimethylamine-N-oxide; d-ROMs, derived reactive oxygen metabolites; OxS, oxidative stress; oxLDL, oxidized LDL.

## Data Availability

The datasets presented in this article are not readily available because the clinical databases are deposited in IDI-IRCCS. Requests to access the databases should be directed to Stefania Madonna, s.madonna@idi.it.

## Supplementary Materials

The following supporting information can be downloaded at: https://www.mdpi.com/article/10.3390/life15111703/s1, Figure S1: Variations in TMAO, d-ROMs, and oxLDL serum levels before and after 16 weeks of therapy with anti-IL-17 or anti-IL-23.; Table S1: Analytical performance parameters of the HPLC-HESI-MS/MS method for TMAO analysis, including linearity range, sensitivity (LOD and LOQ), quality control analytical stability (RSD%); Table S2: Precision and accuracy values of the HPLC-HESI-MS/MS method for TMAO analysis, with measurements of intra- and inter-day precision (CV%) and accuracy (% bias) at different concentrations. References [54,55,56,57,58,59,60,61,62,63] are cited in the supplementary materials.

## Abbreviations

The following abbreviations are used in this manuscript:

BMI	Body mass index
CVR	Cardiovascular risk
DLQI	Dermatology life quality index
d-ROMs	Reactive oxygen metabolites
IL	Interleukins
oxLDL	Oxidized LDL
OxS	Oxidative stress
PASI	Psoriasis area and severity index
TMAO	Trimethylamine-N-oxide
TNF-α	Tumor necrosis factor α
WG	Waist grith
