# Supplementary material for: Anti-IL-17 and Anti-IL-23 Therapies Modulate Serum Biomarkers of Intestinal Dysbiosis and Oxidative Stress Linked to Cardiovascular Risk in Patients with Psoriasis
Source: Life (Basel). 2025 Nov 3;15(11):1703. doi: 10.3390/life15111703 (PMC12653977; doi:10.3390/life15111703)
Supplement: Supplementary file 1 [file life-15-01703-s001.zip › life-3935949-supplementary.pdf]

Supplementary Methods

Determination of TMAO by HPLC-HESI-MS/MS

Serum proteins were precipitated using methanol. Briefly, 50 µL of serum were transferred into 0.5 mL microcentrifuge tubes, followed by the addition of 175 µL of ice-cold methanol. Samples were vortexed for 10 seconds and incubated on ice for 40 minutes to allow complete protein precipitation. The mixtures were then centrifuged at 14,000 × g for 10 minutes at 4 °C. The clear supernatant (~120 µL) was carefully transferred into amber autosampler vials with inserts for subsequent LC–MS/MS analysis.

Quantification of TMAO in serum was performed using a DIONEX UltiMate 3000 HPLC system (Thermo Fisher Scientific, San Jose, CA, USA) equipped with an autosampler, a binary solvent pump, and coupled to an LTQ XL mass spectrometer (Thermo Fisher Scientific, San Jose, CA, USA). Chromatographic separation was achieved on a Luna HILIC column (50 × 2.1 mm, 1.7 µm particle size) fitted with a corresponding HILIC guard column (Phenomenex, Torrance, CA, USA). The mobile phase consisted of (A) 0.15% formic acid and 10 mM ammonium acetate in water, and (B) 100% methanol (LC/MS grade). Separation was carried out in isocratic mode (80:20, A:B) for 6 minutes. The column temperature was maintained at 60 °C, the injection volume was 5 µL, and the flow rate was 0.35 mL/min.

Mass spectrometric detection was performed using a heated electrospray ioniza-tion (HESI) source operating in positive mode with multiple reaction monitoring (MRM). Argon was used as the collision gas at a collision energy of 35.0 eV. The quan-tifier and qualifier MRM transitions for TMAO were m/z 76.1-59.1, and 76.1 – 58.1, re-spectively. MRM parameters were optimized using 1 ppm of methanolic caffeine standard solution.

TMAO quantification was based on a calibration curve ($R^2 \geq 0.99$) constructed from nine different concentrations (2500, 1250, 625, 312.5, 156.2, 78.1, 39, 19.5, and 9.8 ppb), prepared by serial 1:2 dilution and analyzes in triplicate.

The HESI source settings were as follows: sheath gas flow rate, 38; auxiliary gas flow rate, 8; capillary temperature, 320°C; source heater temperature, 130 °C; source voltage, 3.5 kV; source current, 100 µA; capillary voltage, 29 V; and tube lens voltage, 60 V.

Analytical validation of HPLC-HESI-MS/MS method

The HPLC-HESI-MS/MS method was validated according to International Coun-cil for Harmonization (ICH) guidelines [1], including assessment of linearity, limit of detection (LOD), limit of quantification (LOQ), precision, accuracy, analytical stability, and carry-over.

The method's performance is detailed in Table S1, which includes the precision, accuracy, and the results of the linearity study for TMAO. Table S2 further shows the results of the intra- and inter-day precision analyses for TMAO, as well as the sensitivity (LOD and LOQ).

Table S1. Analytical performance parameters of the HPLC-HESI-MS/MS method for TMAO analysis, including linearity range, sensitivity (LOD and LOQ), quality control analytical stability (RSD%)

Compound	Linearity range (ppb)	Calibration curve	R2	LOD (ppb)	LOQ (ppb)	QC (RSD% %)
**TMAO**	10 – 2500	y=4685,1x+74,472	0.992	0.39 ± 0.02	1.29 ± 0.07	3.77

Table S2. Precision and accuracy values of the HPLC-HESI-MS/MS method for TMAO analysis, with measurements of intra- and inter-day precision (CV%) and accuracy (% bias) at different concentrations.

Compound	Concentration (ppb)	Precision (CV%)		Accuracy (% bias)	
		Intraday	Interday	Intraday	Interday
	20	8.12	10.5	-3.52	1.02
	200	1.54	4.89	2.60	0.85

**TMAO**	2000	1.52	3.95	0.66	0.65

The LOD and LOQ were determined to evaluate the sensitivity of the method. The signal-to-noise (S/N) ratio was calculated by comparing the response of samples con-taining known low concentrations of the analyte with that of blank samples [2]. The LOD was defined as the lowest detectable analyte concentration that could be distin-guished from background noise at an S/N ratio of 3. The LOQ was defined as the lowest quantifiable analyte concentration that could be measured with acceptable precision and accuracy, corresponding to an S/N ratio of 10.

Accuracy (expressed as % bias) and precision (expressed as the coefficient of variation, CV%) were determined through intra-day and inter-day analyses of TMAO standard mixtures at three concentration levels (20 ppb, 200 ppb, and 2 ppm). Intra-day precision was assessed by analyzing each concentration three times within a single day, whereas inter-day precision was determined by repeating the analysis over three consecutive days.

To monitor analytical stability throughout the HPLC–HESI–MS/MS sequence, quality control (QC) samples were prepared by pooling aliquots from all biological se-rum samples. QC samples were injected eight times at the beginning of the analytical run to assess platform reproducibility, and subsequently at regular intervals (every four analytical samples) throughout the sequence. For each QC injection, both peak area and retention time were evaluated. Analytical performance was considered acceptable when the relative standard deviation (RSD%) of the peak area for each compound did not exceed 5%, and retention time variation remained within ±0.2 minutes.

All serum samples were analyzed within a single analytical batch to minimize inter-run variability. The injection order was randomized, with groups of four samples analyzed between QC injections (two baseline and two post-treatment samples per group). All analyses were performed in a blinded manner, and the operator remained unaware of sample group allocation (control or treated) throughout the sequence.

Determination of oxidative stress biomarkers

As previously described [3], serum oxidized low-density lipoprotein (ox-LDL) levels were measured using the LP-CHOLOX assay on the automated Free Carpe Diem analyzer (Diacron International, Grosseto, Italy). The commercial kit (lot no. V2503) was used according to the manufacturer's instructions. The LP-CHOLOX test detects lipid peroxidation-derived hydroperoxides, mainly oxidized cholesterol. These hydroperoxides promote the oxidation of ferrous (Fe2+) to ferric (Fe3+) ions, which subsequently react with thiocyanate to form a colored complex quantified spectropho-tometrically at 505 nm. Absorbance values are directly proportional to the lipoperoxide concentration and are calibrated against a standard solution (400 µEq/L). The method demonstrated high precision, with intra- and inter-assay coefficients of variation (CVs) below 2.8%. Results are expressed in µEq/L, with reference intervals defined as follows: normal (≤599 µEq/L), slightly high (600–799 µEq/L), moderately high (800–999 µEq/L), and very high (≥1000 µEq/L) [4,5].

To ensure analytical stability during the colorimetric analysis, quality control (QC) samples were prepared using tert-butyl hydroperoxide (400 µEq/L). QC samples were analyzed six times at the beginning of the analytical sequence to assess platform re-producibility and subsequently at regular intervals (every four analytical samples) throughout the run. The sample analysis order was randomized in groups of four (two baseline and two post-treatment samples) between QC injections. All analyses were conducted in a blinded manner, with the operator unaware of sample group allocation (control or treated).

The total oxidant capacity of serum was assessed using the d-ROMs Lab Test (lot no. V2501; Innovatics Laboratories Inc.), which indirectly quantifies organic hydroperox-ides (ROOH) — major contributors to oxidative potential. The assay is based on the Fenton reaction, in which transition metal ions (iron or copper) catalyze the formation of reactive oxygen species. The oxidation of the chromogenic substrate N,N-diethyl-p-phenylenediamine produces a colored radical cation measured photo-metrically [5-8].

All serum samples were analyzed within a single analytical batch to minimize inter-run variability. Results are expressed in Carratelli units (UCARR), where 1 UCARR corre-sponds to 0.08 mg of hydrogen peroxide (H_2O_2) per 100 mL. The method showed ex-cellent reliability, with intra- and inter-assay CVs below 3.72% for all samples. As previously reported [9,10], d-ROM values are interpreted as follows: normal (250–300 UCARR), borderline (300–320 UCARR), low oxidative stress (321–340 UCARR), moderate oxidative stress (341–400 UCARR), high oxidative stress (401–500 UCARR), and very high oxidative stress (>500 UCARR).

To monitor analytical stability throughout the colorimetric analysis, QC samples were prepared using lyophilized serum with a reference value of 250–300 UCARR. QC sam-ples were analyzed six times at the beginning of the analytical sequence and subse-quently every four analytical samples. The order of analysis and blinding procedures matched those used for the LP-CHOLOX assay.

References

1. Ohno Y. ICH guidelines--implementation of the 3Rs (refinement, reduction, and replacement): incorporating best scientific practices into the regulatory process. *ILAR J.* 2002;43 Suppl:S95-S98. doi:10.1093/ilar.43.suppl_1.s95
2. Maisto M, Schiano E, Novellino E, et al. Application of a Rapid and Simple Technological Process to Increase Levels and Bioccessibility of Free Phenolic Compounds in Annurca Apple Nutraceutical Product. *Foods*. 2022;11(10):1453. Published 2022 May 17. doi:10.3390/foods11101453
3. Barrea L, Muscogiuri G, Pugliese G, et al. Association of Trimethylamine N-Oxide (TMAO) with the Clinical Severity of Hidradenitis Suppurativa (Acne Inversa). *Nutrients*. 2021;13(6):1997. Published 2021 Jun 10. doi:10.3390/nu13061997
4. Macri A, Scanarotti C, Bassi AM, et al. Evaluation of oxidative stress levels in the conjunctival epithelium of patients with or without dry eye, and dry eye patients treated with preservative-free hyaluronic acid 0.15 % and vitamin B12 eye drops. *Graefes Arch Clin Exp Ophthalmol*. 2015;253(3):425-430. doi:10.1007/s00417-014-2853-6
5. Cesarone MR, Belcaro G, Carratelli M, et al. A simple test to monitor oxidative stress. *Int Angiol*. 1999;18(2):127-130.
6. Alberti, A., Bolognini, L., Macciantelli, D. *et al.* The radical cation of *N,N*-diethyl-*para*-phenylendiamine: A possible indicator of oxidative stress in biological samples. *Res Chem Intermed* **26**, 253–267 (2000). https://doi.org/10.1163/156856700X00769
7. Trotti R, Carratelli M, Barbieri M, et al. Oxidative stress and a thrombophilic condition in alcoholics without severe liver disease. *Haematologica*. 2001;86(1):85-91.
8. Gerardi G, Usberti M, Martini G, et al. Plasma total antioxidant capacity in hemodialyzed patients and its relationships to other biomarkers of oxidative stress and lipid peroxidation. *Clin Chem Lab Med*. 2002;40(2):104-110. doi:10.1515/CCLM.2002.019
9. Annunziata G, Ciampaglia R, Maisto M, et al. Taurisolo®, a Grape Pomace Polyphenol Nutraceutical Reducing the Levels of Serum Biomarkers Associated With Atherosclerosis. *Front Cardiovasc Med*. 2021;8:697272. Published 2021 Jul 19. doi:10.3389/fcvm.2021.697272
10. Verde L, Cacciapuoti S, Caiazzo G, et al. Very low-calorie ketogenic diet (VLCKD) in the management of hidradenitis suppurativa (Acne Inversa): an effective and safe tool for improvement of the clinical severity of disease. Results of a pilot study. *J Transl Med*. 2024;22(1):149. Published 2024 Feb 13. doi:10.1186/s12967-024-04853-0

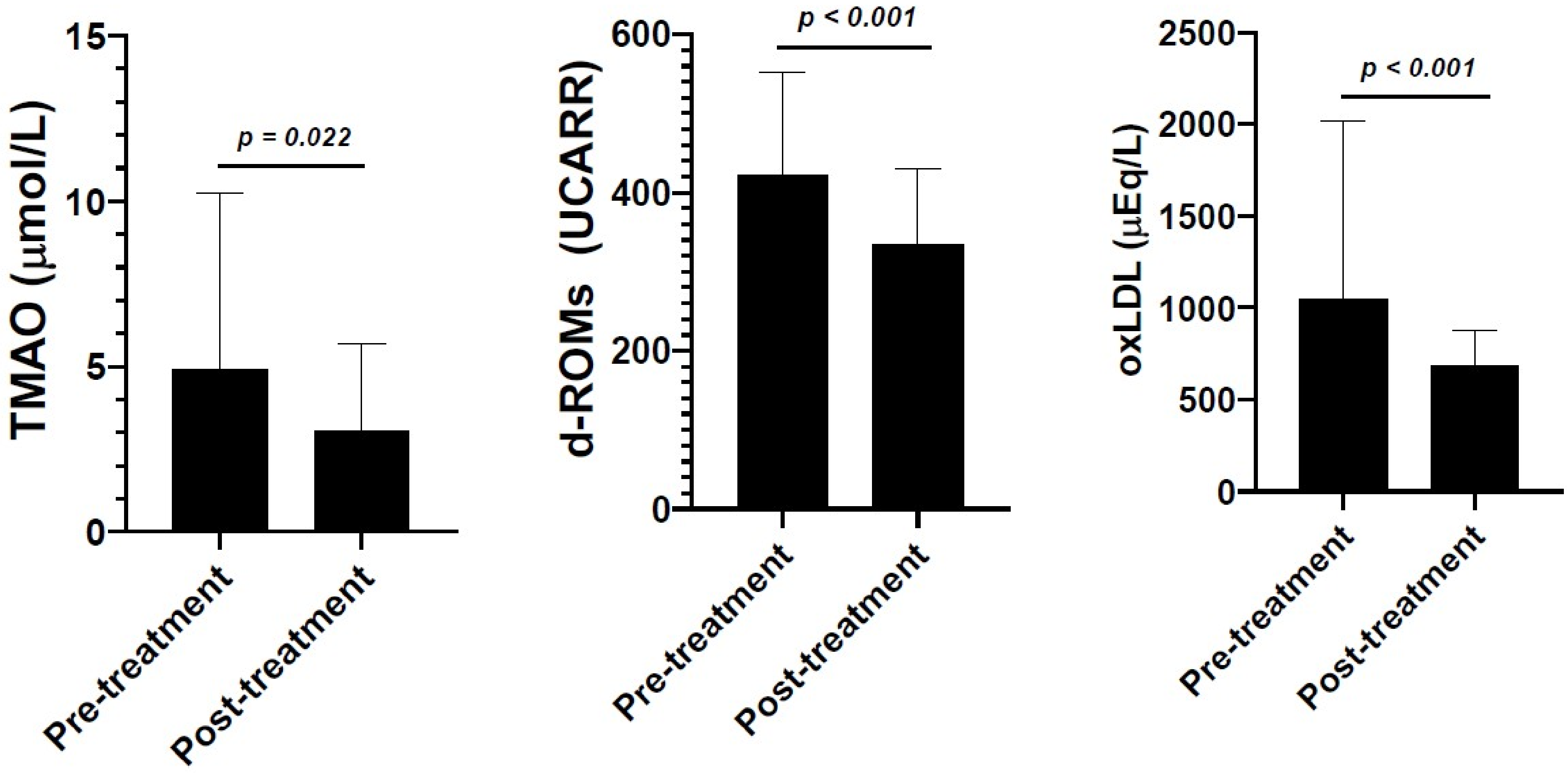

Figure S1. Variations in TMAO, d-ROMs, and oxLDL serum levels before and after 16 weeks of therapy with anti-IL-17 or anti-IL-23. Analyses were performed on the combined cohort of patients receiving either treatment. Data are expressed as mean ± standard deviation (SD). Differences between pre- and post-treatment values were assessed using a paired Student's t test, with statistical significance considered at $p < 0.05$. Abbreviations: interleukin, IL; trimethylamine-N-oxide, TMAO; derived reactive oxygen species, d-ROMs; oxidised LDL, oxLDL.
